# What are the factors affecting older adults’ experience of unmet healthcare needs amid the COVID-19 pandemic in Korea?

**DOI:** 10.1186/s12877-023-04208-2

**Published:** 2023-08-25

**Authors:** Sujin Kim, Jongnam Hwang

**Affiliations:** 1 Korea Institute for Health and Social Affairs, Sejong-si, Korea; 2 Division of Social Welfare & Health Administration, Wonkwang University, Iksandae-ro 460, Jeonbuk, 54538 Korea

**Keywords:** Unmet healthcare need, Older adults, Korea, COVID-19, Health services

## Abstract

**Background:**

Unmet healthcare need is a critical indicator, showing a plausible picture of how the healthcare system works in the unprecedented pandemic situation. It is important to understand what factors affect healthcare services of older adults in the midst of the outbreak, as this could help identify service- and performance-related challenges and barriers to the healthcare system. This study aimed to identify factors associated with unmet healthcare needs among the older Korean population amid the COVID-19 pandemic.

**Methods:**

Cross-sectional data were used from the Experience Survey on Healthcare Use of Older Adults during the COVID-19 (COVID-19 Survey) in Korea (n = 1,917). Our main outcome, unmet healthcare need, was measured based on self-reported experience of overall, regular, and irregular outpatient care services-related unmet healthcare needs. Independent variables were selected based on previous studies on determinants of unmet healthcare need during the COVID-19 pandemic and Andersen’s expanded behavioural model, which theorizes that healthcare-seeking behaviours are driven by psychosocial, enabling, and need factors.

**Results:**

Using multiple logistic regression models, we identified a good understanding of the nation’s health system was associated with lower likelihood of all types of unmet healthcare needs among older Korean adults (OR: 0.39, 95%CI: 0.25–0.61; OR: 0.36, 95%CI: 0.20–0.63; OR: 0.41, 95%CI: 0.23–0.75). Decreased social activities (i.e., shopping and visiting family members) and worsened psychological health issues (i.e., increased anxiety & nervousness and greater difficulty sleeping) were also factors affecting overall and irregular outpatient services-related unmet needs.

**Conclusions:**

To ensure timely access to necessary healthcare services for older adults in the era of the COVID-19 outbreak, improving older adult’s understanding on how the healthcare system works is necessary. Moreover, changes in psychological condition and daily activities due to COVID-19 should be considered as possible barriers to healthcare services among older adults during the global pandemic.

**Supplementary Information:**

The online version contains supplementary material available at 10.1186/s12877-023-04208-2.

## Introduction

Since the initial outbreak of COVID-19 was reported in late 2019, the COVID-19 pandemic has threatened health systems with unprecedented challenges [1]. As of January 2022, all countries around the world still relied on non-pharmacological public health measures, including physical distance, lockdowns, and stay-at-home orders, to flatten the epidemic curve [2]. To control the spread of the virus, implementing such closure measures and providing essential care for COVID-19 patients are necessary, but they result in unexpected consequences, including elevated barriers to accessing health services for health conditions unrelated to COVID-19 [3]. As most health resources are concentrated on dealing with COVID-19-related health issues [4, 5], many people have missed out on necessary care, including regular check-ups, emergency health services, and life-extending interventions [6].

Unmet healthcare need, a spectrum of healthcare need that are not met, has been noted as one of the most worrisome consequences of the COVID-19 pandemic [7]. Unmet healthcare need is an individual’s subjective assessment on whether their needed care is fulfilled or not, and it can arise for a variety reasons, including problem with availability, accessibility, and acceptability. The experience of unmet healthcare need is a pivotal indicator of the effectiveness and efficiency of a nation’s health system and gives a plausible picture of how the health system works, especially in the unprecedented pandemic situation [8]. In general, socio-demographic characteristics are closely associated with reporting unmet healthcare needs. Previous studies highlighted that unmet needs are generally more concentrated in socially and economically vulnerable population groups such as lower income or educational levels, and it is more pervasive among women and those with poor health [9–11]. Since the global pandemic spread, there have been significant reductions in utilization of healthcare services during the pandemic period compared with previous years, and higher rate of unmet healthcare needs were reported in many countries [6, 12]. Accompanied by individual’s socio-demographic factors, the perceived risk of COVID-19-related issues has been identified as a primary barrier to accessing healthcare services primarily in European and North American countries that were severely impacted by the pandemic and implemented strict control measures [13–16]. Given the global pandemic situation, it is crucial to investigate how individual’s perception towards COVID-19 and the changes in their daily lives are also associated with unmet healthcare needs.

Korea is known as one of the countries that successfully limited the spread of COVID-19 and maintained a low mortality rate through strict control measures at the national level [17]. These measures included early detection and the rapid activation of national response protocols led by national leadership. Thus, a robust system was established for the timely diagnostic testing and isolation of the infected individuals. The triage and treatment system were reorganized to handle the influx of COVID-19 patients, complemented by community-based prevention measures such as public awareness campaigns and strict adherence to hygiene practices [18]. The government also mobilized the necessary resources for clinical care without introducing a national lockdown [19]. With respect to the provision of health services in Korea, the expectation was that the required non-emergency health services were not disrupted because the Korea’s universal healthcare system well managed the increasing burden of healthcare need for both COVID-19 and non-COVID-19 patients [20]. However, a recent statistic suggested that the utilization of healthcare services has been reduced compared with the pre-COVID-19 era, and individuals also reported their experience of forgone and delayed care, which suggests that primary or specialty routine care has not been timely provided [21]. Understanding factors associated with unmet healthcare need, particularly those which older adults are experiencing, is an important policy agenda in the midst of the outbreak, and this could help identify service- and performance-related challenges and barriers to the healthcare system [11, 19]. A recent study conducted in Seoul, Korea found that unmet healthcare needs during the pandemic were associated with various socioeconomic factors, including being female, younger, having lower educational levels and higher fear of COVID-19 [22]. While this study provided an initial insight into the factors associated with unmet healthcare needs, it failed to provide a comprehensive snapshot as it did not distinguish the type of healthcare services an individual needed. Furthermore, this study had methodological limitations such as non-nationally representative sample and data collection methods. Given that routine healthcare services have been disrupted in many countries [8], it is also beneficial to understand how factors associated with unmet healthcare needs may vary depending on the type of healthcare services such as regular and irregular services. In particular, it is relevant for older adults who need ongoing healthcare services for existing health issues.

To address these shortcomings of the existing study, this study aimed to examine factors associated with unmet need among the older population in Korea during the COVID-19 pandemic. In order to achieve this, the study utilized a nationally collected data and adopted Andersen’s expanded behavioural model, which can be summarized as the individual characteristics and psychological factors influencing the use of healthcare services.

## Methods

### Data

This study used the Experience Survey on Healthcare Use of Older Adults during the COVID-19 Pandemic (COVID-19 Survey), which was administered to examine the healthcare system’s performance at the national level and the impact of COVID-19 on the healthcare system as a part of the Korea Healthcare System Performance project by the Korea Institute for Health and Social Affairs (KIHASA). The COVID-19 survey yielded cross-sectional data on older adults’ healthcare use experience collected between November 11 and December 5, 2020, in the wake of the COVID-19 outbreak. Eligible participants were over the age of 65 who could communicate in Korean.

Overall, 2,000 survey participants were sampled among a total of 8,684,460 older adults. To ensure a nationally representative sample, the 30 sampling sites were distributed throughout Korea by stratifying 17 metropolitan cities and provinces into 30 regions and then randomly selecting one district in each region. The number of study subjects from each site was allocated in proportion to the square root of the October 2020 population of each region. The participants were chosen based on the gender and age distribution in each region. Supplementary Table 1 displays the distribution of the Korean population and survey participants, highlighting their similarities in terms of sex, age, and region. Face-to-face interviews were performed by trained interviewers using a structured questionnaire and the Computer-Assisted Personal Interviewing (CAPI) methodology. The interviewers entered the answers of each respondent into a computer immediately during the interview, which enabled immediate quality checks and minimized non-responses. The sample size and effective size data were entered into G*Power 3.1.2. software which was used to calculate post hoc analyses of achieved power [23, 24].

The questionnaire was prepared referring to the existing national survey questionnaire. First, the questions on unmet healthcare need, mental health, household income, and social contact changes were referenced from the Survey of Health, Aging, and Retirement in Europe (SHARE) Corona Survey. The questions on self-reported health and chronic diseases were prepared based on the questionnaires from the Korea National Health and Nutrition Survey and the Korea Healthcare Panel. The questions on perceptions of the healthcare system were prepared based on the Korea Healthcare Panel. Lastly, the questions on sociodemographic information were referenced from the SHARE and the Korean Longitudinal Study of Aging (KLoSA). The Institutional Review Board of the Korea Institute for Health and Social Affairs (KIHASA) reviewed and approved the protocol (KIHASA No. 2020-76). Written informed consent was obtained from all participants prior to the survey. Throughout the interviews, adherence to government public health guidelines was followed, including the wearing of facial masks to protect the safety and privacy of the participants.

### Variables

#### Dependent variable: experience of unmet healthcare need

Unmet healthcare need was measured based on self-reported experience. Dependent variables included overall unmet need and unmet healthcare needs for regular and irregular outpatient services, respectively. Since only two individuals reported unmet needs for inpatient care, we included it in the overall assessment of unmet needs but did not specifically analyse unmet needs for inpatient care. Regular outpatient services were defined as clinic or hospital visits to continue receiving treatment for an existing condition such as chronic illness or cancer, while irregular outpatient services were defined as visits due to new health problems. Overall unmet healthcare need was assessed, with the question *“After the COVID-19 outbreak (February 2020), was there ever a time when you felt that you needed healthcare, but you didn’t receive it?”* Eligible responses were *“Yes”* or *“No.”* The respondents who experienced unmet healthcare needs were asked whether regular or irregular outpatient services were delayed and whether the person avoided treatment.

#### Independent variables

Independent variables were selected based on previous studies on determinants of unmet healthcare need during the COVID-19 pandemic and Andersen’s expanded behavioural model, which theorizes that healthcare-seeking behaviours are driven by psychosocial, enabling, and need factors [25, 26]. Psychosocial factors include four main characteristics influencing decision making of planned behaviours including attitudes, knowledge, social norms, and perceived control [27]. Enabling factors are community and individual- level resources related to accessing health services, and individual’s health and functional state are need factors.

In this study, psychosocial factors included individual’s perceptions of the healthcare system, which was measured with a single-select multiple-choice survey for three different indications of perception — perceived understanding, satisfaction, and trust of the nation’s healthcare system. Participants were asked to respond to the following questions: “*How would you rate your understanding, trust, and satisfaction of the healthcare system?*” Possible response options were *“Excellent, very good, good, fair, and poor*”, with the first three options collapsed into “*Good*” and the last two into “*Poor*” for our analyses.

Enabling factors included residence (urban or rural) and household income levels (low, middle or high). Since 299 participants did not respond with the amount and only answered by income category, income was divided into three groups: less than 1 million won (low, approximately 868 US dollars), less than 2 million won (middle), and more than 2 million won (high) referring to previous study [28]. We included self-rated health (good or bad) and chronic condition (none, one, and two or more) as needs factors. Demographic factors included sex (male or female), age (65–69, 70–74, and 75+), marital status (no spouse or with spouse), employment (employed or unemployed), and education level (middle school completion or not). The education level was categorized, considering the distribution.

Considering previous studies on determinants of unmet healthcare need during the COVID-19 pandemic [14, 15, 29], we included COVID-19-related psychosocial health issues, changes in social activities and household income change after the outbreak. For COVID-19-related psychosocial health issue, more anxious & nervous, more sadness & depression, and more sleeping difficulty after COVID-19 were measured as follows. Participants who answered “*Yes*” to the question “*Have you ever been nervous or anxious / been sad or depressed / slept poorly in the past month?*” were asked, “*How is it compared to before the COVID-19 outbreak (January 2020)?*”, with three possible response options of “*I felt less*”, “*I felt no different*,” and “*I felt more.*” The presence of COVID-19-related psychosocial health issues was defined if they answered, “*I felt more*”. For changes in social activities, participants were asked how often they have participated in the following activities compared to before the outbreak – shopping, walking, gathering (with more than five people), and visiting other family members such as relatives. Possible response options were “*Less than before the outbreak, about similar, and more than before the outbreak*”. The COVID-19-related changes in social activities were defined if they answered, “*Less than before the outbreak*”. The decrease in household income after the COVID-19 outbreak was defined as a decrease in the range of one-million-won unit (up to 9 million won in units of 1 million won and over 9 million won) compared to pre-COVID-19 income.

### Statistical analyses

Three separate analyses were conducted to examine factors associated with older Korean adults’ experience of overall unmet healthcare need, unmet healthcare needs for regular and irregular outpatient services, respectively. After 83 participants with missing information were excluded, a total of 1,917 participants were included for the data analyses. Data analyses were performed using STATA v.15, and the results were presented as odds ratio (OR) and 95% confidence interval (CI). Survey weights were applied to all logistic regression analyses.

## Results

Table 1 presents the descriptive characteristics of the 1,917 participants by experiencing overall unmet healthcare need. Of the 171 older adults (8.9%) who reported that they had experienced unmet healthcare need after the COVID-19 outbreak, around half of them experienced unmet healthcare need for regular or irregular outpatient services. Older adults who were unemployed, with highest income, and education below middle school indicated significantly more unmet healthcare need compared to their counterparts. The experience of unmet healthcare need was significantly more common among those reporting poor self-rated health and with COVID-19-related psychosocial health issues such as increased anxiety & nervousness, more sadness & depression, and greater difficulty sleeping. Individuals who went shopping less after the COVID-19 outbreak experienced more unmet need compared to their counterparts. Unmet healthcare need was significantly more common among those who experienced income decrease after the COVID-19 outbreak and who did not have a good understanding of the healthcare system.

**Table 1:** Descriptive characteristics of the study sample by experiencing of overall unmet healthcare need during the COVID-19 outbreak in Korea, 2020 (n = 1,917)

Variables		All		Overall unmet healthcare need			
				Unmet need	No (n = 1,746)	Yes (n = 171)	p-value
		N	%	%	%	%	
Unmet need for regular outpatient services	No	1,828	95.4				
	Yes	89	5.4				
Unmet need for irregular outpatient services	No	1,825	95.2				
	Yes	92	5.6				
Sex	Male	827	43.4	9.3	43.9	39.0	0.25
	Female	1,090	56.6	11.2	56.1	61.0	
Age (years)	65–69	616	32.6	10.0	32.7	31.3	0.62
	70–74	470	24.4	9.4	24.7	22.1	
	75+	831	43.0	11.2	42.6	46.6	
Marital status	With spouse	1,412	74.6	9.7	75.1	70.2	0.18
	No spouse	505	25.5	12.1	25.0	29.8	
Employment	Employed	671	34.9	7.8	35.9	26.2	0.02
	Unemployed	1,246	65.1	11.7	64.1	73.8	
Education	Below middle school	1,044	55.4	14.4	57.4	37.7	< 0.001
	Middle school or above	873	44.7	7.0	42.6	62.3	
Income^†^	Low	971	48.7	9.4	49.1	44.4	0.01
	Middle	434	22.7	7.6	23.4	16.6	
	High	512	28.6	14.1	27.4	39.0	
Residence	Urban	1,387	74.9	10.5	74.7	76.2	0.60
	Rural	530	25.1	9.8	25.3	23.8	
Self-rated health	Good	1,501	78.4	8.9	79.7	67.3	< 0.001
	Bad	416	21.6	15.6	20.4	32.7	
Chronic condition	None	631	33.8	8.2	34.6	26.9	0.15
	1	649	34.3	11.1	34.0	36.8	
	2 or more	637	31.9	11.8	31.3	36.3	
Increased anxiety & nervousness	Not worsened	1,723	89.3	8.7	91.0	74.9	< 0.001
	Worsened	194	10.7	24.3	9.0	25.1	
More sadness & depression	Not worsened	1,710	88.5	9.8	89.1	83.7	0.05
	Worsened	207	11.5	14.7	11.0	16.4	
Greater difficulty sleeping	Not worsened	1,736	90.4	9.0	91.7	79.0	< 0.001
	Worsened	181	9.6	22.6	8.3	21.0	
Decrease in shopping	No decrease	650	33.5	6.7	34.8	21.7	0.001
	Decrease	1,267	66.6	12.2	65.2	78.3	
Decrease in walking	No decrease	840	44.2	16.6	41.1	71.1	< 0.001
	Decrease	1,077	55.8	5.4	58.9	28.9	
Decrease in gathering	No decrease	310	15.6	22.9	13.5	34.6	< 0.001
with 5 or more people	Decrease	1,607	84.4	8.0	86.5	65.4	
Decrease in visits to family members	No decrease	357	18.1	8.6	18.5	15.0	0.30
	Decrease	1,560	81.9	10.7	81.6	85.0	
Income decrease	No	1,379	71.4	8.2	73.1	56.8	< 0.001
	Yes	538	28.6	15.6	26.9	43.2	
Understanding of the healthcare system	No	1,055	55.2	14.2	52.8	75.8	< 0.001
	Yes	862	44.8	5.6	47.2	24.3	
Trust of the healthcare system	No	605	32.1	9.4	32.4	29.0	0.40
	Yes	1,312	67.9	10.8	67.6	71.0	
Satisfaction of the healthcare system	No	539	28.4	11.1	28.2	30.6	0.53
	Yes	1,378	71.6	10.0	71.8	69.4	

^†^Low: less than 1 million won (approx. 868 USD), Middle: less than 2 million won

^¶^ Sampling weights were applied

As shown in Table 2, older adults with a good understanding of the healthcare system showed lower odds of experiencing overall, regular, and irregular outpatient services-related unmet healthcare needs (OR: 0.39, 95%CI: 0.25–0.61; OR: 0.36, 95%CI: 0.20–0.63; OR: 0.41, 95%CI: 0.23–0.75). In addition, older adults who had lower education attainment (OR: 1.90, 95%CI: 1.16–3.10), who had one chronic condition (OR: 1.72, 95%CI: 1.06–2.79), who experienced increased anxiety & nervousness (OR: 3.10, 95%CI: 1.67–5.73) and greater difficulty sleeping (OR: 2.01, 95%CI: 1.12–3.59) after the COVID-19 outbreak were associated with higher likelihood of reporting unmet healthcare need. Thus, those who had decreased their amounts of shopping activity (OR: 3.42, 95%CI: 2.03–5.76), had experienced a decrease in visit to family members after the COVID-19 outbreak (OR: 4.86, 95%CI: 2.33–10.12), and whose household income decreased after the COVID-19 outbreak (OR: 1.66, 95%CI: 1.09–2.52) showed higher odds of experiencing overall unmet healthcare need. By contrast, those who experienced increased sadness and depression, walked less frequently, and had less gathering showed lower odds of overall unmet healthcare need. Unmet healthcare need for regular outpatient services was higher among older adults who were unemployed (OR: 2.33, 95%CI: 1.11–4.87), with lower educational attainment (OR: 2.34, 95%CI: 1.27–4.29), and who had chronic conditions (OR: 13.54, 95%CI: 4.12–44.42; OR: 13.99, 95%CI: 4.17-47.00). The participants who had decreased their amounts of shopping reported more likelihood of experiencing regular outpatient services-related unmet need (OR: 2.20, 95%CI: 1.17–4.14). Those who walked less frequently reported lower odds of regular outpatient services-related unmet need. Unmet need for irregular outpatient services was higher among those who had lower education attainment (OR: 2.68, 95%CI: 1.25–5.75), who felt increased anxiety & nervousness (OR: 4.91, 95%CI: 2.41–9.99) in addition to those with greater difficulty sleeping after the COVID-19 outbreak (OR: 2.35, 95%CI: 1.06–5.21). Regarding the COVID-19-related changes in social activities, older adults who spent less their time for shopping (OR: 3.31, 95%CI: 1.76–6.23) and who less visited their family members (OR: 4.39, 95%CI: 2.04–9.47) show a higher likelihood of reporting irregular services-related unmet healthcare need. Those who had low income, experienced increased sadness and depression, walked less frequently, and had less gathering showed lower odds of overall unmet healthcare need. Unweighted logistic regression revealed a significant relationship in multiple covariates (Table S3).

**Table 2:** Odds ratio and 95% Confidence interval from logistic regression models examining factors associated with unmet healthcare need among older Korean adults (n = 1,917)

Variables		Overall		Regular services			Irregular services		
		OR	95%CI		OR	95%CI		OR	95%CI
Sex	Male	Ref.			Ref.			Ref.	
	Female	0.99	(0.66–1.48)		0.70	(0.43–1.14)		0.96	(0.54–1.73)
Age (years)	65-69	Ref.			Ref.			Ref.	
	70-74	0.67	(0.38–1.18)		0.74	(0.36–1.52)		0.52	(0.23–1.17)
	75+	0.69	(0.40–1.19)		0.57	(0.28–1.18)		0.85	(0.40–1.81)
Marital status	With spouse	Ref.			Ref.			Ref.	
	No spouse	1.07	(0.69–1.68)		1.16	(0.68–1.97)		0.77	(0.40–1.50)
Employment	Employed	Ref.			Ref.			Ref.	
	Unemployed	1.34	(0.83–2.17)		2.33^*^	(1.11–4.87)		1.11	(0.58–2.11)
Education	Below middle school	1.90^*^	(1.16–3.10)		2.34^**^	(1.27–4.29)		2.68^*^	(1.25–5.75)
	Middle school or above	Ref.			Ref.			Ref.	
Income^†^	Low	0.64	(0.39–1.04)		0.60	(0.33–1.09)		0.47^*^	(0.23–0.97)
	Middle	0.60	(0.36–1.01)		0.66	(0.33–1.34)		0.63	(0.33–1.22)
	High	Ref.			Ref.			Ref.	
Residence	Urban	Ref.			Ref.			Ref.	
	Rural	0.88	(0.60–1.28)		0.87	(0.52–1.46)		0.95	(0.59–1.52)
Self-rated health	Good	Ref.			Ref.			Ref.	
	Bad	1.41	(0.91–2.18)		1.25	(0.77–2.03)		1.63	(0.83–3.21)
Chronic condition	None	Ref.			Ref.			Ref.	
	1	1.72^*^	(1.06–2.79)		13.54^***^	(4.13–44.42)		0.68	(0.35–1.33)
	2 or more	1.60	(0.95–2.68)		13.99^***^	(4.17-47.00)		0.53	(0.25–1.11)
Psychological health issues	Increased anxiety & nervousness	3.10^***^	(1.67–5.73)		1.69	(0.83–3.43)		4.91^***^	(2.41–9.99)
	More sadness & depression	0.43^*^	(0.20–0.92)		0.80	(0.36–1.79)		0.21^**^	(0.08–0.57)
	Greater difficulty sleeping	2.01^*^	(1.12–3.59)		1.34	(0.67–2.68)		2.35^*^	(1.06–5.21)
Social activity	Less shopping	3.42^***^	(2.03–5.76)		2.20^*^	(1.17–4.14)		3.31^***^	(1.76–6.23)
	Less walking	0.26^***^	(0.17–0.39)		0.41^***^	(0.25–0.68)		0.20^***^	(0.11–0.38)
	Less gathering	0.10^***^	(0.05–0.18)		0.46	(0.19–1.08)		0.07^***^	(0.04–0.14)
	Less visits to family members	4.86^***^	(2.33–10.12)		2.19	(0.83–5.79)		4.39^***^	(2.04–9.47)
Decreased income	Yes	1.66^*^	(1.09–2.52)		1.69	(0.99–2.91)		1.46	(0.83–2.54)
Understanding of the healthcare system	Good	0.39^***^	(0.25–0.61)		0.36^***^	(0.20–0.63)		0.41^**^	(0.23–0.75)
Trust of the healthcare system	Good	1.39	(0.86–2.25)		1.38	(0.73–2.58)		1.40	(0.72–2.75)
Satisfaction of the healthcare system	Good	0.86	(0.53–1.39)		1.16	(0.59–2.26)		0.72	(0.39–1.34)

***p < 0.001, **p < 0.01, * p < 0.05, CI Confidence Interval

^†^Low: less than 1 million won (approx. 868 USD), Middle: less than 2 million won

^¶^ Sampling weights were applied

## Discussion

In the midst of the COVID-19 pandemic era, identifying which factors affect unmet healthcare need, particularly among older adults who typically have higher demands regarding healthcare services, is important for policy makers to respond to the need to develop an effective healthcare system. The findings from our study contribute to understanding multiple factors that influence older adults’ experience of unmet healthcare need, by adapting Andersen’s expanded behavioural model and COVID-19-related determinants of unmet healthcare need, using the nationally collected survey dataset. Our findings indicated that older adults with a good understanding of the nation’s healthcare system had less likelihood of experiencing all types of unmet healthcare needs. In addition, the experience of overall unmet healthcare need was less likely to apparent among those with self-reported worsened psychological health issues after the pandemic, such as increased anxiety & nervousness and greater difficulty sleeping. Lastly, older adults who reported less participation in shopping activities and less visiting their family members after the pandemic were more likely to experience overall and irregular outpatient-related unmet healthcare needs.

Among various psychosocial factors, an individual’s understanding of the nation’s healthcare system, particularly how well the older adults know the nation’s system, is closely linked to unmet healthcare need. This finding suggests that old adults who are well-oriented to the current health system may not feel any barriers to access or difficulties in using healthcare services. A good understanding of the health system could be interpreted as an individual’s perception, and previous studies have argued that good perception of the surrounding physical and social environment facilitates or limits the utilization of necessary healthcare services [30–32]. For instance, urban residents who had poor perceptions of the healthcare-related environment or health system were more likely to report unmet healthcare need [31]. Vulnerable population groups, for example a refugee who has limited understanding or knowledge of the healthcare system in the country in which they have recently settled, often expressed difficulties in seeking healthcare services because they are not familiar with the system itself and were unsure of where to receive the needed health services [33]. In this sense, our result, a close link between understanding of the healthcare system and unmet healthcare need, implies that better understanding of the healthcare system facilitates older adults’ access and utilization of the necessary health services for both regular and irregular services, regardless of the chaotic pandemic situation. Meanwhile, it is worthwhile to note that trust and satisfaction in the health system did not appear to be related to any unmet healthcare need. This might suggest that understanding of the health system is a better indicator for determining how well the health system works under the current circumstances.

Worsened psychological health issues after the pandemic, such as anxiety & nervousness and sleep difficulty have been thought to be risk factors for experiencing unmet healthcare need in the COVID-19 era, and our findings are in line with previous studies [22, 34]. Accumulative studies highlighted the importance of mental health condition for unmet healthcare need under the current pandemic situation [22, 35]. It has been suggested that psychological stressors negatively affect individual’s health-seeking behaviours so that older adults who need care for their worsened psychological health conditions become reluctant to access or use healthcare services [36]. It is plausible that older Korean adults with worsened psychological health issues after the pandemic wanted to seek the relevant health services for their issues but failed to receive the needed care as the COVID-19 pandemic continued. It is interesting to note that while higher anxiety and nervousness and sleep difficulty were positively associated with older adult’s experience of unmet healthcare needs, sadness and depression was negatively associated with unmet needs, particularly for overall and irregular services. Our findings are in contrast to the previous finding, suggesting that depressive symptoms are closely linked to higher likelihood of experiencing unmet healthcare needs [37]. This may imply that older adults with more sadness or depression may have no or limited healthcare needs because experience of unmet healthcare need is subjective assessment at the individual level. In fact, previous study suggested that individuals experienced frequent feeling of sadness reported higher healthcare avoidance [38]. In this sense, our findings could be interpreted that older adults with more sadness and depression due to COVID-19 may be inclined to avoid or not actively seek irregular outpatient services, ultimately resulting in a deterioration of their health condition.

There has been increasing interest in the role of social activity on unmet healthcare need beyond individual characteristics [39]. Previous studies suggested that an individual’s social networks and social activities are positively correlated with healthcare accessibility and utilization [39–41]. Older adults with frequent contacts with other individuals such as relatives and friends could strengthen their social network, allowing for greater information exchange to better cope with their health issues and improving adherence to healthcare appointments [42, 43]. In line with previous studies, our results, which indicated less participation in shopping and less visiting family members after the global outbreak, suggest that decreased social activity and social interaction due to the fear of COVID-19 infection or adherence to public health measures may induce limited access to and less use of healthcare services by avoiding or cancelling non-emergency and necessary services [3, 19]. It is interesting to note that older adults who less participated in walking and gathering activities were correlated with a lower probability of experiencing overall and irregular outpatient services-related unmet healthcare needs. It is unclear why the impacts of different social activities on unmet healthcare need vary. A plausible explanation is that older adults’ adherence to the COVID-19 public health measures may contribute to reduction or unrecognition of healthcare needs themselves, particularly irregular outpatient services because the public health measures limit daily outdoor activities and social gatherings. To fully understand the relationship between type of social activities and unmet healthcare need, further investigation is needed.

Furthermore, it has been reaffirmed that lower educational attainment and chronic conditions are key factors associated with experiencing unmet healthcare need among older Korean adults. Similar findings have been reported in previous studies [10]. Existing studies also reported that older people may have higher healthcare need or use more medical services due to their disabilities and chronic health conditions, poor socioeconomic status, or social isolation [3, 15, 44]. Not receiving needed health services could be life-threatening and result in death as an outcome, especially for older adults [45–47]. To prevent the worst health outcome in the older adult population, it is necessary to develop policy interventions to tackle difficulties with access to health services among older adults with lower educational attainment and chronic conditions.

In addition to psychosocial factors, economic status, measured by a decline in household income after the COVID-19 outbreak, has been a considerable factor affecting the experience of unmet healthcare need. In previous studies from the pre-pandemic period, lower income was a major determinant of experiencing unmet healthcare need [9, 48, 49]. Interestingly, a decrease in income after the pandemic plays a more important role in unmet healthcare need than lower income itself among older adults in Korea. This is in line with previous studies reporting the impact of income loss on unmet healthcare need among the Korean population [10, 50]. Although health coverage continues to expand and healthcare costs continue to be subsidized for older adults, additional strategies might need to be considered to diminish financial hardship to healthcare services.

Lastly, it is worth noting that the rate of unmet healthcare needs in our study was relatively low, approximately 5% among total respondents, compared to previously reported rates ranging from approximately 8–17% [10, 51]. The reason for this low rate for unmet is inconclusive, but it is possible that the perceived need for healthcare services among older adults was significantly decreased during the COVID-19 pandemic, leading to underreporting of healthcare needs. To fully understand the reasons behind this low reported rate of unmet healthcare needs, further studies need to be considered.

### Limitations

Despite several meaningful findings of our study, there are several limitations that should be mentioned. Due to the nature of cross-sectional data, the causality between various factors and unmet healthcare need among older Korean older adults were not able to further investigate. In addition, it should be noted that other factors, for instance clinical needs and provider characteristics that were not included in our study could also influence older adults’ experience of unmet healthcare needs during the COVID-19 pandemic. In addition, the reasons for unmet healthcare need cannot be distinguished. It needs to be considered to collect further information including reasons for and frequency of unmet healthcare need as the COVID-19 pandemic is becoming endemic [52]. The COVID-19 survey we used in this study was collected older adults’ information based on rigorous sampling approaches to ensure national representation; however, it is still possible that older adults with a greater fear of COVID-19 infection may have avoided participating in the face-to-face survey interview.

## Conclusions

Because of the universal healthcare system and the successful strict infection measures in Korea, it was expected that the required non-COVID-19 related health services were not disrupted. However, the results from our study found that older adults still reported their experience of unmet healthcare need, in particular a good understanding of the national healthcare system was associated with lower likelihood of experiencing unmet healthcare needs regardless of types of healthcare services. In addition, our study found that the COVID-19-related changes in social activities and income were important factors associated with older adults’ experience of unmet healthcare need for regular and irregular outpatient services in Korea in addition to worsened psychological health issues. To ensure timely access to necessary healthcare services for older adults in the era of the COVID-19 outbreak, improving older adult’s understanding on how the healthcare system works is necessary. Moreover, changes in psychological condition and daily activities due to COVID-19 should be considered as possible barriers to healthcare services among older adults during the global pandemic.

### Electronic supplementary material

Below is the link to the electronic supplementary material.

Supplementary Material 1

## Data Availability

The data that support the findings of this study are available from Korea Institute for Health and Social Affairs (https://data.kihasa.re.kr), but restrictions apply to the availability of these data, which were used under license for the current study, and are not publicly available. Data are, however, available from the authors upon reasonable request and with permission of Korea Institute for Health and Social Affairs.

## List of abbreviations

CAPI: Computer-Assisted Personal Interviewing
KIHSA: Korea Institute for Health and Social Affairs
SHARE: Survey of Health, Aging, and Retirement in Europe
OR: Odds Ratio
