# Supplementary material for: What are the factors affecting older adults’ experience of unmet healthcare needs amid the COVID-19 pandemic in Korea?
Source: BMC Geriatr. 2023 Aug 25;23:517. doi: 10.1186/s12877-023-04208-2 (PMC10463954; doi:10.1186/s12877-023-04208-2)
Supplement: Supplementary file 1 — Supplementary Material 1 [file 12877_2023_4208_MOESM1_ESM.docx]

**Supplementary material**

Table S1. Sex, age, and region distribution of the Korean population and survey participants

Table S2. Descriptive characteristics without weights

Table S3. Odds ratio and 95% Confidence interval from logistic regression models without weights

|  |  | Male |  |  | Female |  |
| --- | --- | --- | --- | --- | --- | --- |
|  | 65-69 | 70-74 | 75+ | 65-69 | 70-74 | 75+ |
| Korean population |  |  |  |  |  |  |
| 1. Seoul | 2.91 | 2.18 | 3.03 | 3.31 | 2.57 | 4.39 |
| 2. Busan | 1.28 | 0.91 | 1.18 | 1.44 | 1.07 | 1.86 |
| 3. Daegu | 0.75 | 0.53 | 0.72 | 0.86 | 0.64 | 1.22 |
| 4. Incheon | 0.82 | 0.57 | 0.73 | 0.85 | 0.63 | 1.23 |
| 5. Gwangju | 0.36 | 0.28 | 0.38 | 0.42 | 0.34 | 0.63 |
| 6. Daejeon | 0.41 | 0.29 | 0.39 | 0.44 | 0.32 | 0.63 |
| 7. Ulsan | 0.33 | 0.21 | 0.22 | 0.33 | 0.21 | 0.37 |
| 8. Sejong | 0.07 | 0.05 | 0.06 | 0.07 | 0.05 | 0.11 |
| 9. Gyeonggi | 3.44 | 2.38 | 3.36 | 3.62 | 2.70 | 5.35 |
| 10. Gangwon | 0.58 | 0.38 | 0.67 | 0.59 | 0.44 | 1.11 |
| 11. Chungcheongbuk | 0.53 | 0.36 | 0.57 | 0.55 | 0.39 | 0.99 |
| 12. Chungcheongnam | 0.68 | 0.53 | 0.85 | 0.71 | 0.57 | 1.45 |
| 13. Jeollabuk | 0.63 | 0.52 | 0.77 | 0.66 | 0.59 | 1.39 |
| 14. Jeollanam | 0.68 | 0.56 | 0.88 | 0.69 | 0.64 | 1.70 |
| 15. Gyeongsangbuk | 1.01 | 0.73 | 1.11 | 1.07 | 0.80 | 2.05 |
| 16. Gyeongsangnam | 1.10 | 0.76 | 1.02 | 1.15 | 0.85 | 1.95 |
| 17. Jeju | 0.18 | 0.14 | 0.20 | 0.20 | 0.16 | 0.37 |
| Survey participants |  |  |  |  |  |  |
| 1. Seoul | 2.95 | 2.20 | 3.05 | 3.30 | 2.55 | 4.40 |
| 2. Busan | 1.30 | 0.90 | 1.20 | 1.45 | 1.05 | 1.85 |
| 3. Daegu | 0.75 | 0.55 | 0.70 | 0.85 | 0.65 | 1.20 |
| 4. Incheon | 0.80 | 0.55 | 0.75 | 0.85 | 0.65 | 1.25 |
| 5. Gwangju | 0.35 | 0.30 | 0.40 | 0.40 | 0.35 | 0.65 |
| 6. Daejeon | 0.40 | 0.30 | 0.40 | 0.45 | 0.30 | 0.65 |
| 7. Ulsan | 0.35 | 0.20 | 0.20 | 0.35 | 0.20 | 0.35 |
| 8. Sejong | 0.05 | 0.05 | 0.05 | 0.05 | 0.05 | 0.10 |
| 9. Gyeonggi | 3.45 | 2.35 | 3.35 | 3.60 | 2.70 | 5.35 |
| 10. Gangwon | 0.60 | 0.40 | 0.65 | 0.60 | 0.45 | 1.10 |
| 11. Chungcheongbuk | 0.55 | 0.35 | 0.60 | 0.50 | 0.40 | 0.95 |
| 12. Chungcheongnam | 0.65 | 0.55 | 0.85 | 0.75 | 0.55 | 1.45 |
| 13. Jeollabuk | 0.60 | 0.55 | 0.80 | 0.65 | 0.60 | 1.40 |
| 14. Jeollanam | 0.70 | 0.55 | 0.85 | 0.70 | 0.65 | 1.70 |
| 15. Gyeongsangbuk | 1.00 | 0.75 | 1.10 | 1.05 | 0.80 | 2.05 |
| 16. Gyeongsangnam | 1.10 | 0.75 | 1.00 | 1.15 | 0.85 | 1.95 |
| 17. Jeju | 0.20 | 0.15 | 0.20 | 0.20 | 0.15 | 0.35 |

Table S1. Sex, age, and region distribution of the Korean population as of the end of October and survey participants

Table S2. Descriptive characteristics of the study sample without weight by experiencing of overall unmet healthcare need during the COVID-19 outbreak in Korea, 2020 (n=1,917)

|  | | All | | Overall unmet healthcare need | | |
| --- | --- | --- | --- | --- | --- | --- |
| Variables | |  |  | No (n= 1746) | Yes (n= 171) | p-value |
|  | | N | % | % | % |  |
| Unmet need for regular services | No | 1,828 | 95.4 |  |  |  |
|  | Yes | 89 | 4.6 |  |  |  |
| Unmet need for irregular services | No | 1,825 | 95.2 |  |  |  |
|  | Yes | 92 | 4.8 |  |  |  |
| Sex | Male | 827 | 43.1 | 43.6 | 38.0 | 0.16 |
|  | Female | 1090 | 56.9 | 56.4 | 62.0 |  |
| Age (years) | 65 – 69 | 616 | 32.6 | 32.2 | 31.6 | 0.44 |
|  | 70 - 74 | 470 | 24.4 | 24.9 | 21.1 |  |
|  | 75+ | 831 | 43.0 | 43.0 | 47.4 |  |
| Marital status | With spouse | 1412 | 74.5 | 74.1 | 69.6 | 0.21 |
|  | No spouse | 505 | 25.5 | 26.0 | 30.4 |  |
| Employment | Employed | 671 | 34.9 | 35.7 | 27.5 | 0.03 |
|  | Unemployed | 1246 | 65.1 | 64.3 | 72.5 |  |
| Education | Below middle school | 1044 | 55.4 | 56.2 | 36.3 | <0.001 |
|  | Middle school or above | 873 | 44.6 | 43.8 | 63.7 |  |
| Income^☨^ | Low | 971 | 48.6 | 51.2 | 45.0 | 0.01 |
|  | Middle | 434 | 22.7 | 23.1 | 18.1 |  |
|  | High | 512 | 28.6 | 25.7 | 36.8 |  |
| Residence | Urban | 1387 | 74.9 | 73.0 | 65.5 | 0.04 |
|  | Rural | 530 | 25.1 | 27.0 | 34.5 |  |
| Self-rated health | Good or fair | 1501 | 21.6 | 79.3 | 68.4 | 0.001 |
|  | Bad | 416 | 78.4 | 20.7 | 31.6 |  |
| Chronic condition | None | 631 | 33.8 | 33.5 | 27.5 | 0.28 |
|  | 1 | 649 | 34.3 | 33.6 | 36.3 |  |
|  | 2 or more | 637 | 31.9 | 32.9 | 36.3 |  |
| Increased anxiety & nervousness | Not worsened | 1723 | 89.3 | 91.3 | 75.4 | <0.001 |
|  | Worsened | 194 | 10.7 | 8.7 | 24.6 |  |
| More sadness & depression | Not worsened | 1710 | 88.5 | 89.7 | 84.2 | 0.03 |
|  | Worsened | 207 | 11.5 | 10.3 | 15.8 |  |
| Greater difficulty sleeping | Not worsened | 1736 | 90.4 | 91.6 | 79.5 | <0.001 |
|  | Worsened | 181 | 9.6 | 8.4 | 20.5 |  |
| Decrease in shopping | No decrease | 650 | 33.4 | 35.2 | 21.1 | <0.001 |
|  | Decrease | 1267 | 66.6 | 64.8 | 79.0 |  |
| Decrease in walking | No decrease | 840 | 44.2 | 41.4 | 68.4 | <0.001 |
|  | Decrease | 1077 | 55.8 | 58.6 | 31.6 |  |
| Decrease in gathering | No decrease | 310 | 15.6 | 14.4 | 33.9 | <0.001 |
| with 5 or more people | Decrease | 1607 | 84.4 | 85.6 | 66.1 |  |
| Decrease in visits to family members | No decrease | 357 | 18.1 | 19.1 | 13.5 | 0.07 |
|  | Decrease | 1560 | 81.9 | 80.9 | 86.6 |  |
| Income decrease | No | 1379 | 71.4 | 73.5 | 56.1 | <0.001 |
|  | Yes | 538 | 28.6 | 26.5 | 43.9 |  |
| Understanding of the | No | 1055 | 55.0 | 53.0 | 76.0 | <0.001 |
| healthcare system | Yes | 862 | 45.0 | 47.0 | 24.0 |  |
| Trust of the | No | 605 | 31.6 | 32.0 | 27.5 | 0.23 |
| healthcare system | Yes | 1312 | 68.4 | 68.0 | 72.5 |  |
| Satisfaction of the | No | 539 | 28.1 | 27.9 | 30.4 | 0.49 |
| healthcare system | Yes | 1378 | 71.9 | 72.1 | 69.6 |  |

^☨^Low: less than 1 million won (approx. 868 USD), Middle: less than 2 million won; 171 individuals experienced overall unmet needs.

Table S3. Odds ratio and 95% Confidence interval from logistic regression models without weights examining factors associated with unmet healthcare need among older Korean adults (n=1,917)

| Variables | | Overall | | Regular services | | | Irregular services | | |
| --- | --- | --- | --- | --- | --- | --- | --- | --- | --- |
|  |  | OR | 95%CI | | OR | 95%CI | | OR | 95%CI |
| Sex | Male | Ref. |  | | Ref. |  | | Ref. |  |
|  | Female | 1.02 | (0.69-1.51) | | 0.79 | (0.48-1.29) | | 1.14 | (0.66-1.96) |
| Age (years) | 65－69 | Ref. |  | | Ref. |  | | Ref. |  |
|  | 70－74 | 0.61 | (0.35-1.05) | | 0.76 | (0.38-1.53) | | 0.45* | (0.20-0.99) |
|  | 75+ | 0.64 | (0.38-1.08) | | 0.59 | (0.30-1.17) | | 0.73 | (0.36-1.47) |
| Marital status | With spouse | Ref. |  | | Ref. |  | | Ref. |  |
|  | No spouse | 1.06 | (0.69-1.63) | | 1.22 | (0.73-2.05) | | 0.68 | (0.36-1.28) |
| Employment | Employed | Ref. |  | | Ref. |  | | Ref. |  |
|  | Unemployed | 1.27 | (0.81-1.99) | | 1.83 | (0.98-3.44) | | 1.09 | (0.60-1.98) |
| Education | Below middle school | 2.06** | (1.33-3.20) | | 2.41** | (1.36-4.28) | | 2.91*** | (1.56-5.42) |
|  | Middle school or above | Ref. |  | | Ref. |  | | Ref. |  |
| Income^☨^ | Low | 0.6* | (0.38-0.95) | | 0.55* | (0.31-0.98) | | 0.46* | (0.24-0.88) |
|  | Middle | 0.65 | (0.39-1.08) | | 0.68 | (0.34-1.36) | | 0.67 | (0.34-1.31) |
|  | High | Ref. |  | | Ref. |  | | Ref. |  |
| Residence | Urban | Ref. |  | | Ref. |  | | Ref. |  |
|  | Rural | 1.41 | (0.95-2.09) | | 1.29 | (0.78-2.13) | | 1.48 | (0.88-2.50) |
| Self-rated | Good or fair | Ref. |  | | Ref. |  | | Ref. |  |
| health | Bad | 1.39 | (0.90-2.15) | | 1.32 | (0.80-2.17) | | 1.6 | (0.84-3.04) |
| Chronic  condition | None | Ref. |  | | Ref. |  | | Ref. |  |
|  | 1 | 1.71* | (1.05-2.81) | | 12.14*** | (4.11-35.87) | | 0.69 | (0.36-1.31) |
|  | 2 and more | 1.41 | (0.84-2.36) | | 11.61*** | (3.88-34.75) | | 0.47* | (0.23-0.94) |
| Psychological  health issues | Increased anxiety & nervousness | 3.13*** | (1.79-5.46) | | 1.96 | (0.98-3.92) | | 4.23*** | (2.04-8.77) |
|  | More sadness & depression | 0.45* | (0.23-0.89) | | 0.85 | (0.38-1.86) | | 0.18** | (0.07-0.51) |
|  | Greater difficulty sleeping | 1.92* | (1.06-3.47) | | 1.29 | (0.63-2.66) | | 2.45* | (1.09-5.55) |
| Social activity | Less shopping | 3.6*** | (2.17-5.99) | | 2.42** | (1.26-4.67) | | 4.13*** | (2.11-8.06) |
|  | Less walking | 0.29*** | (0.20-0.44) | | 0.41*** | (0.25-0.67) | | 0.22*** | (0.12-0.40) |
|  | Less gathering | 0.09*** | (0.05-0.17) | | 0.44* | (0.20-0.97) | | 0.06*** | (0.03-0.13) |
|  | Less visits to family members | 5.89*** | (3.00-11.58) | | 2.67* | (1.08-6.61) | | 5.52*** | (2.42-12.60) |
| Decreased income | Yes | 1.72** | (1.15-2.58) | | 1.62 | (0.97-2.70) | | 1.64 | (0.95-2.84) |
| Understanding of the healthcare system | Good | 0.37*** | (0.24-0.55) | | 0.37*** | (0.22-0.64) | | 0.36*** | (0.20-0.65) |
| Trust of the healthcare system | Good | 1.61* | (1.02-2.55) | | 1.53 | (0.85-2.75) | | 1.62 | (0.85-3.08) |
| Satisfaction of the healthcare system | Good | 0.83 | (0.53-1.31) | | 1.05 | (0.58-1.89) | | 0.7 | (0.37-1.31) |

***p<0.001, **p<0.01, * p<0.05, CI Confidence Interval

^☨^Low: less than 1 million won (approx. 868 USD), Middle: less than 2 million won
